# The Anatomy of Onomatopoeia

**DOI:** 10.1371/journal.pone.0028317

**Published:** 2011-12-14

**Authors:** María Florencia Assaneo, Juan Ignacio Nichols, Marcos Alberto Trevisan

**Affiliations:** Laboratory of Dynamical Systems, Physics Department, University of Buenos Aires, CABA, Buenos Aires, Argentina; University of Buenos Aires, Argentina

## Abstract

Virtually every human faculty engage with imitation. One of the most natural and unexplored objects for the study of the mimetic elements in language is the onomatopoeia, as it implies an imitative-driven transformation of a sound of nature into a word. Notably, simple sounds are transformed into complex strings of vowels and consonants, making difficult to identify what is acoustically preserved in this operation. In this work we propose a definition for vocal imitation by which sounds are transformed into the speech elements that minimize their spectral difference within the constraints of the vocal system. In order to test this definition, we use a computational model that allows recovering anatomical features of the vocal system from experimental sound data. We explore the vocal configurations that best reproduce non-speech sounds, like striking blows on a door or the sharp sounds generated by pressing on light switches or computer mouse buttons. From the anatomical point of view, the configurations obtained are readily associated with co-articulated consonants, and we show perceptual evidence that these consonants are positively associated with the original sounds. Moreover, the pairs vowel-consonant that compose these co-articulations correspond to the most stable syllables found in the knock and click onomatopoeias across languages, suggesting a mechanism by which vocal imitation naturally embeds single sounds into more complex speech structures. Other mimetic forces received extensive attention by the scientific community, such as cross-modal associations between speech and visual categories. The present approach helps building a global view of the mimetic forces acting on language and opens a new venue for a quantitative study of word formation in terms of vocal imitation.

## Introduction

One controversial principle of linguistics is the arbitrariness of the linguistic sign [1], which can be roughly described as the lack of links between the acoustic representation of the words and the objects they refer to. Besides the specific implications of this principle in language and language evolution, there is a class of words located on the verge of the problem: the onomatopoeic words, which are already embedded in the phonetic space and linked to the objects they name by imitative forces. This unique linguistic condition has also a neural counterpart: recent investigations show that onomatopoeic sounds are processed by extensive brain regions involved in the processing of both verbal and no-verbal sounds [2].

From the diverse forms of mimicry in the animal kingdom to virtually every high human function, imitation is a fundamental biological mechanism generating behavior [3]. An approach to the imitative components of language is therefore a challenging question that has been cast aside, due in part to the very different acoustical properties of non-human sounds like collisions, bursts and strikes compared to the string of vowels and consonants forming their onomatopoeias.

Here we address this question by defining vocal imitation as the transformation of a sound into the ‘best possible’ speech element, the one that minimizes their spectral difference within the anatomical constraints of the vocal system. We make this definition operational using a mathematical model for voice generation based on anatomical parameters. In the early history of voice production models, mechanical artifacts mimicking the vocal system served to identify the physical principles underlying the generation of voice and to postulate phenomenological descriptions for more complex vocal phenomena [4]. In the last two decades, the approach of dynamical systems took hold. The motivation behind working with mathematical models is the convenience of framing the basic physical mechanisms of voice production in simple mathematical terms, and working out the anatomically related parameters that could easily be compared with experimental ones. This point of view quickly showed its benefits: the use of dynamical models served to map complex acoustical properties of the sounds to the physiological and anatomical constraints of the vocal system [5]–[7] and, far beyond its original aim, it also allowed elucidating the neural structure behind vocal production in songbirds [8], [9], extending the original problem to a global understanding of the vocal production and neural control in biological systems.

**Figure 1:**
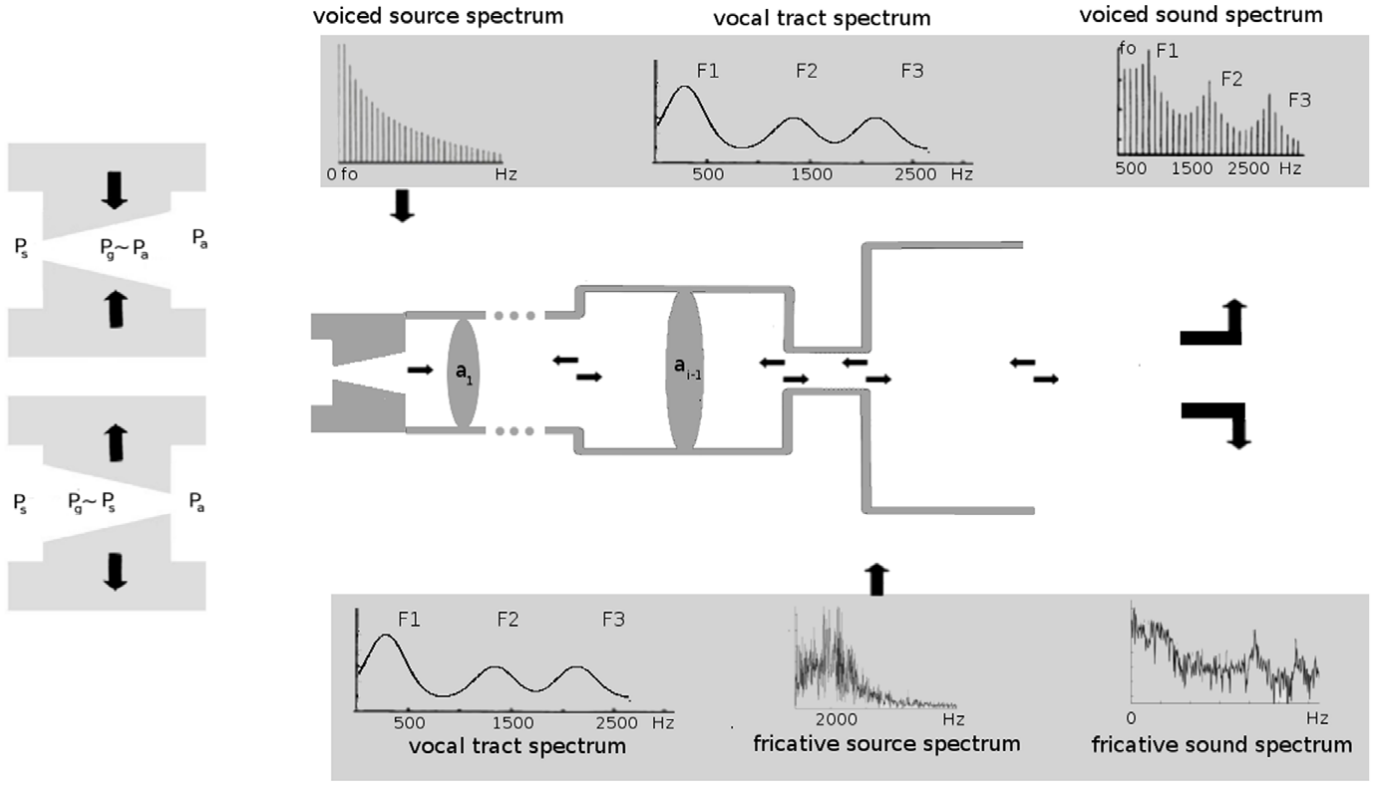
Sketch of the vocal model. The figure in the middle represents the concatenation of tubes that approximate the vocal tract. The upper panel represents, from left to right, the voiced source spectrum of fundamental frequency 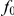, the vocal tract transfer function for a tube of about 17.5 cm and the multiplication of both, corresponding to the resulting voiced sound. In the lower panel, a colored noise sound source characterizing the turbulent flow at the exit of the constriction at the section 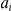 of the vocal tract and the resulting fricative sound, filtered by the vocal tract.

In this work we aim at showing that the dynamical approach is also a pertinent tool to investigate the role of vocal imitation in word formation. The human vocal system is incapable of generating exact copies of a given sound. It is constrained both by the anatomy and physiology of the human vocal system and by the phonetic space of the speakers' native language that shapes the sounds that are better produced and perceived. Roughly, the vocal system consists of two main blocks: the glottis (enclosing the vocal folds), connected upstream to the vocal tract, a set of articulated cavities that extends from the glottal exit to the mouth. These two blocks are usually identified with the *sound production* and the *sound filtering* respectively. While this is essentially true for the filtering process, that basically depends on the vocal tract, there are two main ways in which speech sounds can be generated by the vocal system, giving rise to *voiced* and *unvoiced* sounds respectively. A sketch of the vocal production system is displayed in figure 1.

*Voiced* sounds are generated as airflow perturbations produced by the oscillating vocal folds are injected into the entrance of the vocal tract. The principle behind sustained oscillation without vocal tract coupling is shown schematically in figure 1. The vocal folds change their profile during an oscillation cycle, in such a way that pressure acting on them (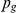) approaches sub-glottal pressure 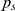 (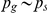) during the opening cycle with a convergent profile, and the vocal tract pressure 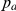 (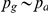) during the closure characterized by a divergent profile. In normal conditions, 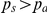 and therefore a net energy transfer occurs from the airflow to the vocal folds. In [10], a dynamical system depending on biological parameters is described for the fold dynamics of songbirds, relying on the described principle. Here we use it as the sound source for voiced sounds, adapting its parameters to the human system (see Methods). The resulting oscillations are characterized by a spectrally rich signal of fundamental frequency 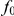 and spectral power 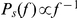, as sketched in figure 1 (upper panel, left).

This signal travels back and forth along the vocal tract, which is identified with a non-uniform open-closed tube, characterized by a smooth transfer function 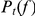 with peaks on the resonant frequencies 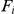, called formants. The formant frequencies are perturbations of the formants for a uniform tube, which for a tube of length 17.5 cm are located at 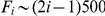 Hz for positive integers 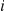 (figure 1, upper panel, middle). We approximate this tube as a concatenation of 10 short uniform tubes of total length 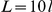 and cross sections 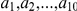 (figure 1, middle panel). At each interface, transmitted and a reflected sound waves are created, and their interference pattern creates a speech sound whose spectrum is sketched in figure 1, right upper panel.

On the other hand, *unvoiced* sounds are produced in many different ways. In particular, fricative consonants are produced when air encounters a narrow region of the vocal tract, generating a turbulent jet downstream the constriction (as sketched in figure 1, lower panel, middle). Unlike voiced sounds, source-filter separability does not hold for turbulent sound sources [4], [11]. Here we propose a very simple model for these fricatives as a colored noise source located at the exit of a constriction, centered in 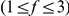 kHz and variable width (see Methods).

The complete model of vocal fold dynamics, turbulent sound source and sound propagation through the vocal tract allows synthesizing a variety of speech sounds from a set of anatomical parameters. However, in this work we deal mainly with the inverse problem. Given a target spectrum 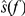, we want to recover the anatomical parameters 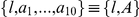 of the vocal system that produced it, which imply searching in a multidimensional parameter space and fitting the results in the frequency range where the model holds (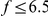 kHz for plane wave propagation [4], [11]). In these conditions, the mapping from the spectral to the anatomical space is not one-to-one, and many different vocal anatomies will be compatible with a given speech sound. In order to deal with this variability, we set up a genetic algorithm that, working together with the model, allows an efficient exploration of the parameter space and returns a family of vocal tracts compatible with the experimental spectrum (see Methods).

Throughout this work, we use this model to explore anatomic features of sounds of different complexity, from vowels and simple fricative consonants to the vocal configurations that imitate non-speech sounds of nature.

## Results

### Vowels and fricative consonants

**Figure 2:**
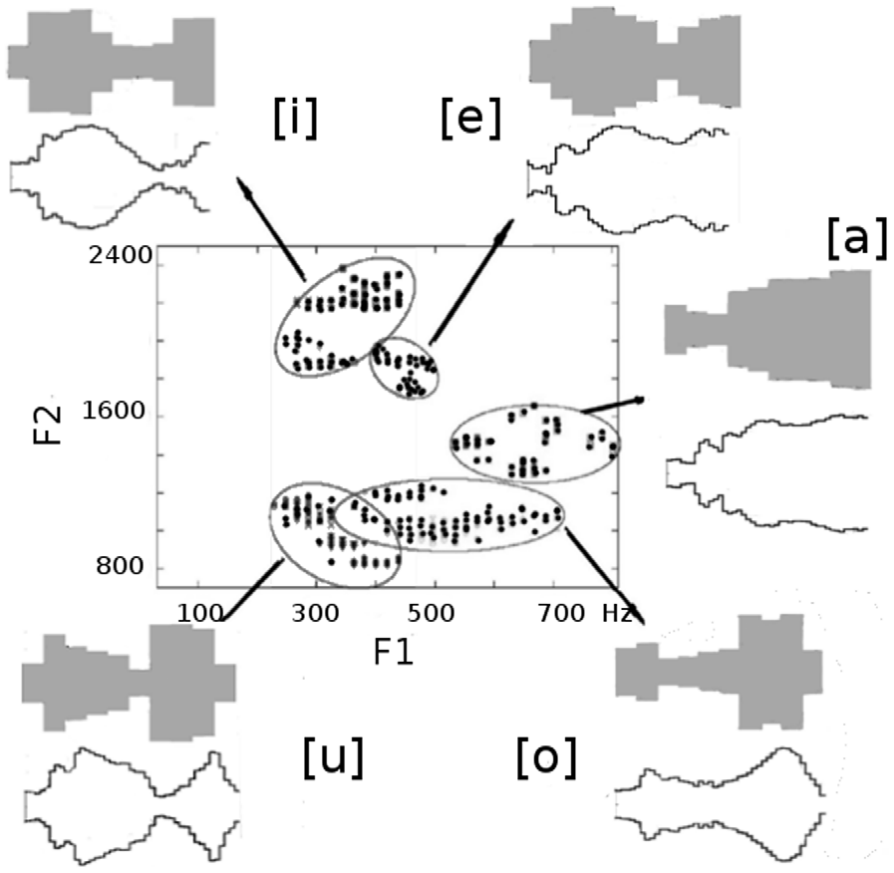
Anatomy of vowels. Each point in the graph corresponds to a vowel sample (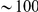 ms) taken from normal speech recordings of 20 Spanish speakers of different age and sex. We performed a Fast Fourier Transform to the time series to get the vowel spectrum and plot the first two formants 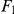 and 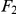. The points naturally cluster into five groups, associated with the Spanish vowels 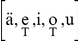. The figures defined by the black lines are vocal tract shapes taken from a corpus of MRI-based anatomical data reported in [12]. In each case, we selected from the corpus the vowels that were closer, from a phonetic point of view, to the Spanish vowels: 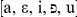. MRI-based data consists of 44 area functions taken from equally spaced slices of vocal tract shapes 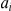, 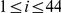. The shapes drawn here correspond to the solid of revolution of radius 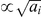. On the other hand, the gray shapes are the reconstructed vocal tracts from our model (see Methods).

One of the most striking properties of vowels is that they can be characterized by the first two vocal tract resonances, the formants 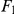 and 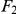, regardless of any other acoustic feature. This is the origin of the standard vowel representation that we reproduce in figure 2, where we show 40 speech samples from 12 speakers pronouncing the 5 Spanish vowels 
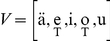
, that sound like the bold part of the words t**i**me, pl**ay**, fr**ee**, c**oa**t and b**oo**t respectively. Clearly, in this space the samples are clustered in 5 distinct groups.

For each group, two vocal tract shapes are shown. The contours defined by black lines are selected from a corpus of MRI-based vocal tract shapes for English speakers reported in [12]. We show vocal tracts for 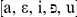, which are the most similar to the set of Spanish vowels from a phonetical point of view.

The gray shapes are the vocal tracts retrieved by our model, proceeding as follows: first, we select 10 utterances for each vowel of a speaker in our bank. We calculate their spectra and use the average as a target spectrum for our model, from which we retrieve a family of different 10-tube vocal tracts producing sound spectra compatible with the target spectrum (up to 5% error, see Methods). In figure 2 we show, for each vowel, an average over that family of 10-tube vocal tracts.

One of the advantages of our model is that it automatically generates a diversity of anatomical solutions compatible with a given experimental speech spectrum. Interestingly, if just the information of the two first formants is used to fit the model parameters, a variety of different vocal tract shapes is obtained. When spectral information is used in the whole range 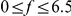 kHz, which roughly include the first 4 formants, the resulting vocal tracts converge to more stable configurations, with low dispersion from the average (gray shapes of figure 2).

The anatomical differences that appear between the reconstructed and MRI-based vocal tracts can be due to interpersonal anatomical differences, and to pronunciation differences. Some experimental MRI-data for a subset of Spanish vowels is available [13] displaying better agreement with our reconstructed vocal tracts. However, for the sake of consistency, we compare our vowels with the more complete corpus of experimental vocal tract data reported in [12].

We further tested our results with a perceptual experiment. We synthesized sounds using the 5 reconstructed vocal tracts for vowels (files S1, S2, S3, S4 and S5 for vowels 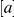, 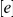, 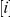, 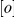 and 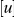 respectively, see Supplementary Information) and asked 20 subjects to freely associate a vowel to the audio files (see Methods). The results, compiled in the table 1, show that synthetic sounds generated with the reconstructed vocal tracts are consistently associated with the original vowels.

**Table 1:** Matrix of associations between synthesized sounds and vowels.

	A	E	I	O	U
**A**	20	0	0	0	0
**E**	0	17	2	1	0
**I**	0	2	16	0	2
**O**	2	0	0	18	0
**U**	0	0	0	4	16

Associations between vowels (first row) and synthesized sounds (first column) for 20 participants. The sounds were synthesized using the anatomical parameters of table 2 for the 5 Spanish vowels, as displayed in figure 2, and fixed source parameters (see Methods). The incorrectly associated audio files correspond mainly to neighboring vowels in the 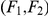 space (see figure 2).

Next, we explored the anatomy of voiceless fricative consonants. Examples of these consonants are [f, 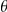, s, 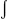, ç, x], that sound like the bold part of the words **f**ace, **th**in, **s**tand, **sh**eep, **h**ue and lo**ch** respectively. In this case, sound is created by the turbulent passage of air through a constriction of the vocal tract. The listed consonants are ordered according to their constriction location down into the vocal tract, from the lips up to the velum. We simulate the fricatives using a simple colored noise source located at the exit of the constriction, which propagates along the vocal tract (see Methods). Given a vocal tract configuration, the only condition imposed by the model is that turbulence occurs at the exit of the narrowest tube.

**Figure 3:**
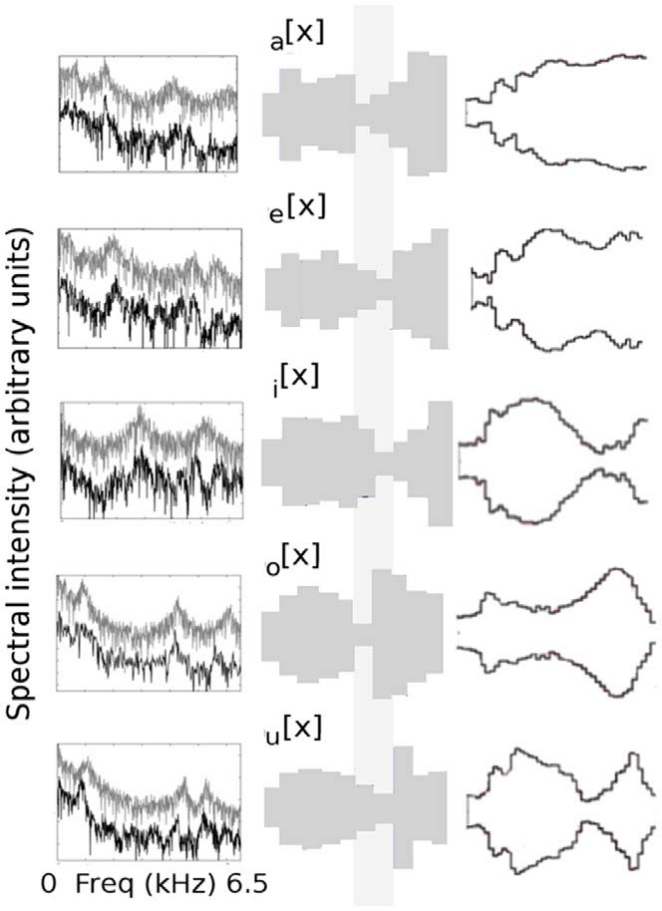
Co-articulated fricatives. From top to bottom, reconstructed vocal tract configurations of co-articulated fricatives 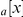, 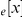, 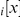, 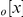 and 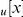 (gray shapes) and their associated MRI vowel data [12] (black contours). The obtained shapes are a combination of the preceding vowel and a constriction at the velar level (located around half the vocal tract length), indicated by the watermark. These vocal tract configurations along with the source parameters 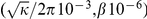 are: 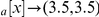, 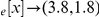, 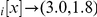, 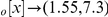, 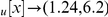 generate sounds having the spectra in black, to be compared with the experimental spectra, in gray.

We explored the vocal anatomy of 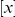 in different vocalic contexts, using experimental recordings of the vowel-consonant pairs 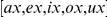 and 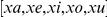. The case is interesting because, during speech, articulatory gestures are partially inherited from one phoneme to the other and therefore the configuration for the fricative consonant is expected to carry signatures of both sounds [14]. In order to study the anatomical signatures of the missing vowels, we extracted exclusively the consonant part from the audio files, calculated their spectra and use them as the target spectra for our model. The results are summarized in figure 3 and table 2, where again we show the vocal tracts of fricative 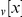 together with the MRI data for vowel 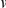 that coarticulate with them. As expected, every vocal tract systematically displays a constriction at the velar level (gray watermark of figure 3), which is the anatomical signature of the consonant 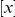 [12] and the overall shape of their correspondent neighboring vowels.

**Table 2:** Average diameters and lengths for the 10-tube vocal tract approximations.

	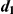	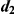	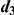	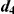	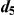	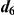	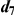	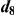	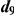	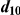	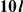
					cm						cm
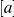	1.00	0.72	0.62	1.58	2.03	2.48	2.46	2.49	2.84	2.89	16.4
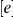	0.76	1.35	1.92	1.95	1.64	1.43	0.65	1.23	1.52	1.65	16.4
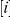	0.84	2.39	2.42	2.45	1.85	0.95	0.86	0.71	1.32	1.48	16.4
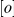	1.21	1.48	0.68	0.79	0.98	1.12	2.96	2.64	3.00	1.09	16.4
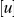	1.23	2.74	0.40	1.64	1.70	2.09	1.79	1.70	1.85	2.04	17.4
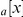	1.15	0.50	1.13	1.00	1.46	0.48	1.65	2.43	2.76	2.89	16.4
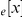	0.67	1.22	0.93	1.29	0.85	0.62	0.31	1.25	1.53	1.95	16.4
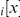	0.59	1.77	1.72	1.65	1.80	1.40	0.45	0.76	1.21	2.45	16.4
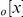	1.14	1.90	2.26	2.02	1.72	0.37	3.00	2.90	2.12	1.91	16.4
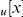	0.80	1.73	1.78	1.49	1.33	0.98	0.61	2.74	1.40	1.60	17.4
click	0.86	1.43	1.86	1.73	1.70	1.72	0.22	1.72	1.54	2.45	16.4
knock	0.57	1.59	1.84	1.29	1.31	1.34	0.65	0.32	3.00	0.69	16.4
click (anat.)	0.75	1.81	1.42	1.39	1.56	1.19	0.23	1.54	1.44	2.45	16.4
knock (anat.)	0.54	2.02	1.88	1.08	2.12	0.43	2.45	1.21	2.10	1.07	16.4

The anatomical parameters of the vocal tracts retrieved by our model for vowels and fricative consonants. The last rows correspond to the best imitations for the click and the knock sounds (without and with anatomical restrictions). We show the diameters 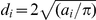 for the 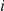-th tube and total length 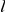.

Although consonants effectively inherit anatomic properties of their neighboring vowels, the relative order of the pair (preceding or succeeding vowel) does not appreciably affect the anatomy of the consonant. Throughout this work, we identify a consonant co-articulated with a vowel 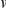 with a subscript 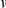 in front of the consonant, regardless of the vowel context.

### Onomatopoeia

Onomatopoeias aim at imitating sounds produced by people, animals, nature, machines and tools. The last three categories are particularly challenging for imitation, as sounds are not produced by another vocal system and therefore imply strong imitative efforts. Here we will specifically deal with the sounds that come from striking blows on doors and pressing light switches or computer mouse buttons, which are also readily associated with the English onomatopoeias *knock* and *click*. These, in turn, are well established words that, in their present form, have a long tradition, dating from at least 8 and 4 centuries ago respectively.

From a phonetic point of view, the click-type onomatopoeia typically presents slight variations across languages, usually in the form of suffixes. This is probably due to its association with technological gadgets used worldwide and certainly we cannot conclude from its stability the action of language-independent imitative forces. Some other forms are also present, like the Spanish *tic*, of homologous use. The case of the knock-type onomatopoeia is different, with more dispersion across languages, as in the examples of table 3. Two remarks are in order here: first, there are very stable subsets of speech elements across languages (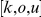 for the knock-type and 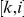 for the click-type). Second, these subsets are not disjoint: for instance, 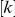 is a very stable element shared by both type of onomatopoeias.

**Table 3:** Onomatopoeias associated with the action of knocking across languages.

Language	Action	Onomatopoeia
Spanish	Golpear	to**k**
Italian	Bussare	to**k**
French	Frapper	to**k**
English	To knock	no**k**
German	Klopfen	**k**lopf
Polish	Pukak	pu**k**
Japanese	Takete	**k**on
Dutch	Kloppen	**k**lop
Hungarian	kopogtato	**k**op
Bulgarian	bluskam	chu**k**
Thai	kor	**k**o**k**

The listed onomatopoeias were recorded from native speakers (we use approximate English pronunciations). Notably, the consonant 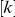 is present in every language in either context 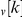 or 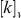 for the vowels 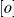 and 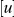. Many other examples of the knock onomatopoeia are available on the Internet, for instance at the wikipedia 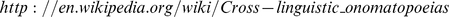, where very few exceptions to this rule are reported. It is interesting to note that some languages allow the onomatopoeic sounds to permeate into related nouns and verbs, while in others they are completely different. It has been suggested that onomatopoeias, which are mainly monosyllabic, are more permeable to languages with the same predominance, as the case of English.

**Figure 4:**
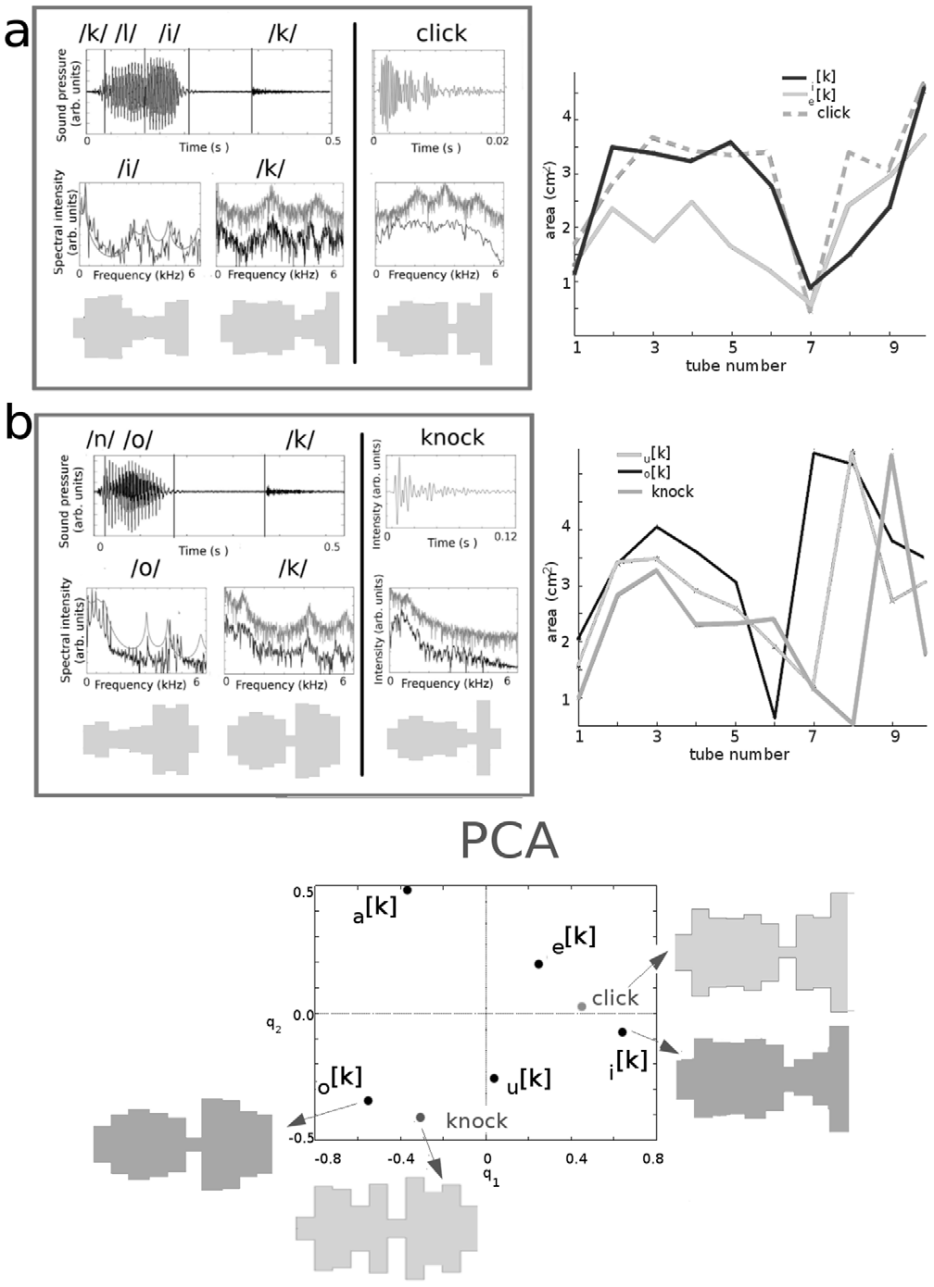
Anatomy of onomatopoeias. We compare sound time series, spectra and anatomy of the click (panel a) and knock (panel b) onomatopoeias and their corresponding sounds. As evident from the time series for the knock and click words (upper insets), the occlusive consonants 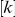 are naturally isolated from the rest of the speech sounds during the pronunciation of the onomatopoeias in normal speech. However, co-articulation strongly affects their spectral content (medium insets): the occlusive consonants 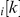 and 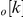 consist of superimposing a velar constriction on a vocal tract that globally resembles the vowels 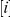 in click and 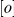 in knock (lower insets). The figures to the right within the frame represent the *best* vocal tracts imitating the click and knock sounds as retrieved by our model, without anatomical restrictions. To the right, outside the frame, we show the area functions for the occlusive consonants 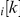 (black) and 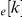 (gray) for the click (dotted) and 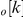 (black) and 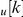 (light gray) for the knock (gray). In the bottom panel we show the first two components 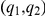 of the PCA for the co-articulated consonants and best imitations: 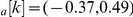; 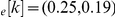; 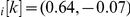; 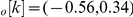; 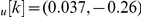; knock = 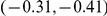 and click = 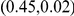. The distances between the knock vocal tract and the coarticulated consonants are: 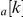 = 0.90; 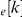 = 0.82; 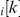 = 1.00; 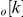 = 0.26; 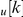 = 0.38. The distances between the click vocal tract and the coarticulated consonants are: 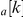 = 0.95; 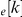 = 0.26; 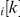 = 0.21; 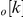 = 1.07; 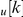 = 0.50.

On the other hand, the sounds associated with these onomatopoeia are acoustically very different. Knocks are short sounds characterized by a convex decaying spectral intensity that becomes negligible around 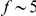 kHz, while click-type sounds are even shorter sounds displaying a concave spectral intensity, distributed in the range 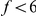 kHz. These properties, shown in figure 4, are very stable for the noises falling under these two onomatopoeic classes (see Methods, Natural sounds).

In order to compare speech with non-speech sounds, we hypothesize that imitative speech sounds try to optimize their spectral content with respect to the original sounds. We focus on spectral information for many reasons. First, because from the very first stage of the auditory processing, the inner ear performs a form of mechanical Fourier transform along the cochlea, revealing that spectral information is essential to hearing. Second, because here we are not dealing directy with onomatopoeias as words, but instead with imitative elements within them, and whereas word identification strongly depends on the speech envelope, important information of non-speech sounds is encoded in its fine structure [2], [15]. Finally, because different speech sounds can be treated as the same in the spectral domain. For instance, the plosive consonant 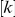 (as in the bold part of **k**iss) is produced by the sudden pressure liberation occurring when opening a completely occluded vocal tract, generating a fast increase and a bit slower decay of the sound intensity. Notably, the location of the tract occlusion for 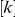 coincide with the constriction point for the fricative consonant 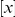, and both sound sources are considered analogous [4]. Moreover, the spectra of both consonants are almost indistinguishable for time frames of 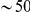 ms, the stable part of the plosive. Here we neglect the very short initial burst of the plosive and simulate the 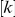 as the stationary fricative 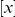 multiplied by its sound envelope, thus recovering in a simple way most of the spectral and temporal features of both speech sounds. In the following, we use the plosive 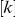 in the place of the fricative 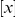 unless further clarification is needed.

Within this paradigm of vocal imitation, we run our model using knocks and clicks as target spectra. The results for both cases are compiled in the two frames of figure 4, where we show the time series of the onomatopoeia and its related sound (upper inset), the spectra of the most representative vowel and consonant and the sound spectrum (middle inset) and their reconstructed anatomic configurations (lower inset).

The classic features that describe the vocal tract from a phonetic-articulatory point of view are the aperture of the jaw, the position of the tongue and the roundedness of the lips [4]. The first two features are loosely related to the relative size and place of the tube with maximal cross section, while the third is more tightly related to the relative areas of the last tubes (open or closed). With respect to these descriptive features, the click vocal tract share with 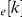 and 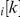 the unroundedness of the lips, and 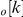 and 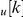 share the lip rounding with the knock vocal tract. Beyond this qualitative description, there are some anatomical discrepancies between the co-articulated consonants and the best imitations. In particular, the shapes of the best imitations seem more sharp than the consonants. Since our vocal model do not impose any constraints to the reconstructed vocal tracts, the anatomical plausibility of these vocal tracts must be examined. In [12], Story finds that any experimental vocal tract of area 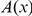, can be very well approximated by 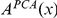, with 
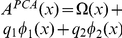
 for proper coefficients 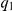 and 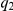. Here, 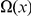 is a neutral vocal tract and 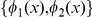 the two first eigenmodes of the principal component analysis (PCA), calculated over a corpus of 10 different vowels. In this way, the anatomical restrictions imposed by the vocal articulators can be accounted for in an elegant mathematical manner. Following this idea, we include anatomical information in our fitness function, penalizing the difference 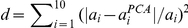 between a given vocal tract of areas 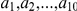 and its approximation using the first two most significant components (see Methods, Genetic algorithm). In this work, we performed the principal component analysis (as described in [16]) using our set of vowels and fricative consonants. The best imitations for clicks and knocks subjected to these restrictions are shown in the two dimensional space of the most significant components 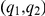 (bottom panel of figure 4). In this space, the imitative vocal tracts are clearly closer to 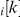 and 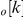 for the click and knock sounds respectively.

Based on these results at the level of voice production, we also explored the imitative components of onomatopoeia from a perceptual point of view, in two different experiments. In both of them, participants were instructed to listen to a series of audio files without any information about the nature of the sounds they were about to listen. They had to evaluate their similarity with respect to their own representation of striking a blow on a door, using a scale from 1 (no association) to 10 (perfect identification). In another session, the participants repeated the experiment but this time they evaluated the similarity of the audio files with the sound of pressing on a light switch/computer mouse button.

**Figure 5:**
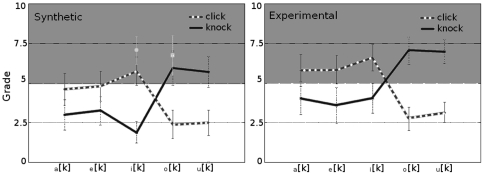
Associations between co-articulated consonants, knocks and clicks. We evaluate the similitude of 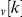 sounds with respect to the knock (solid line) and click (dotted line) sounds. Participants graded the audio files using a scale from 1 (poor or no association) to 10 (perfect identification). The left panel summarizes the responses of 20 participants to 7 synthetic sounds: the 5 co-articulated 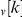, using the parameters of 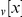 (figure 3 and table 2) modulated by an experimental 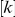 envelope (see Methods). The other 2 sounds were generated using the best vocal tracts for the knock and click sounds, modulated by the same 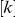 envelope (points in light gray). The stronger associations with the click and knock sounds are 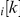 and 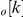 respectively. The best vocal tracts performed better than the consonants. In the right panel, we show the results of the experiment for 20 subjects using experimental isolated fricatives 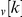. The trend is the same as before, but grades are systematically higher.

In the first experiment (see Methods), they listened to 5 experimental records of isolated consonants 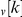 in random order (two sets of experimental audio files are also available at Supporting Information, Audio S6, S7, S8, S9, S10 and S11 a S15 ordered as 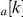, 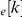, 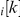, 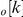 and 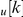 for each set). The average grades obtained for the 20 participants are shown in right panel of figure 5: the dotted line corresponds to associating the consonants with the light switch sound, and the solid line to associations with the strike on a door. The two groups 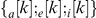 and 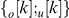 form two well separate clusters (Wilcoxon test 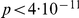 for the click and 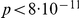 for the knock associations). Although differences between consonants within each cluster do not reach significance, the strongest association with the click sound corresponds to 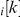, with an average grade of 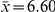 (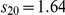). The best association with the knock sound is 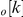, 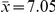 (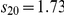).

In the second experiment, 20 different subjects listened to 7 synthetic recordings of the 5 reconstructed consonants 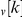 and the best vocal configurations for the click and knock sounds (audio available at Supporting Information, Audio S16, S17, S18, S19 and S20 for 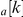, 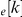, 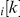, 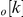 and 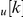 respectively, S21 and S22 for the optimal knock and click). Results are summarized in the left panel of figure 5. Although milder, we found curves showing the same trends as in the previous case, but average grades systematically lower. We remark that our model for fricative and plosive sounds is mainly designed to capture the basic spectral features of the consonants analyzed here and lacks specific features that are important from the perceptual point of view. Therefore synthetic sounds generated with our model are insufficient to reproduce the results obtained with experimental unvoiced sounds. Nevertheless, the best grades still correspond to the synthetic 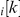 with 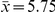 (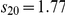) and 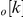 with 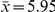 (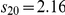). Moreover, the synthetic sounds generated with the best imitative vocal tracts (light gray points) are perceived as closer to the original sounds than the consonants (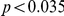), with 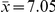 (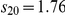) for the click and 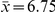 (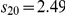) for the knock.

These results suggest that the most stable speech sounds within the knock and click onomatopoeias across languages are indeed linked to the sounds they refer to by imitation. We provide evidence of this connection from both the voice production and perception levels. From the point of view of speech production, the vocal configurations of the coarticulated consonants 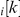 and 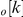 approach the configurations that maximize the acoustical similitude to the click and knock sounds within the constraints of the vocal system. On the other hand, from a purely perceptual point of view, these speech sounds, isolated from the word context, are positively associated with the original sounds, showing that both the unvoiced sound and the neighbouring voiced sound, even if this last is missing, are necessary for imitative purposes in onomatopoeia. In the next section we discuss this particular role of the co-articulation in the production of onomatopoeias.

## Discussion

In a recent work, Chomsky pointed out that the striking human ability of vocal imitation, which is central to the language capacity, has received insufficient attention [17]. As a matter of fact, although scarce, specific literature about onomatopoeias provides definitive evidence in favor of its pertinence in the study of imitation and language [2]. In this work we study the existence of pure imitative components in two types of onomatopoeia. The controversy posed by onomatopoeia is that one could ideally expect that the imitation of a simple noise should be a single speech sound, the closest one from an acoustical point of view. However, as any other word, onomatopoeias are formed by strings of speech sounds of very different properties, v.g. vowels and consonants.

Although seemingly irreconcilable, both perspectives can be approached in terms of *co-articulation*. On one hand, we showed that the best imitations of click and knock sounds are close, in the the anatomical space, to the configurations of co-articulated consonants. In fact, our experiments show evidence that the isolated speech sounds 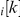 and 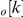 elicited strong associations with knock and click sounds. Even though the instructions probably dragged their attention to noises, when asked, the participants did not recognize the files as speech sounds. This is notable, considering that subjects perform good at complex tasks with similar stimulae, as recognizing missing vowels from co-articulated fricatives [14]. Globally, our results help supporting the idea that part of the onomatopoeic structure is in fact driven by imitation and that the speech sounds that maximize the acoustic similarity with respect to the original noises correspond to simple speech sounds.

On the other hand, co-articulated sounds naturally refer to their constitutive vowel-consonant pairs, therefore linking a single sound to a syllabic structure. Notably, both 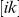 and 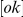 are the most stable syllables of the analyzed onomatopoeias across languages, suggesting that these syllables are natural units in the onomatopoeic formation. In this way, a picture appears in which vocal imitation of single sounds deploys into a more complex structure of different sounds: vowels that help achieving the correct spectral load and give sonority to the onomatopoeia, and stop consonants that account for the noisy content and provide for the correct temporal features of the sound.

Nevertheless, this explanation does not exhaust the problem of onomatopoeic formation. As any other word with a long tradition, onomatopoeias contain elements accumulated across history, elements beyond pure acoustic imitation [18]. It is well known that mild, universal forms of synaestesia participate in speech structures. In particular, visual cues like shape, size and brightness affect the speech sounds used to name objects [19]. Therefore, a complete explanation of the onomatopoeic structure should include cross-modal relationships and their interaction with vocal imitation. We believe that this perspective, merging physical modeling of the vocal system and perceptual experiments, will help building a global picture of the basic mimetic forces acting on word formation.

## Methods

### Ethics statement

A total of 40 native Spanish speakers (24 females and 16 males, age 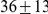) with normal hearing participated in the experiments and signed a written consent form. All the experiments described in this paper were reviews and approved by the ethics comittee: “Comité de Ética del Centro de Educación Médica e Investigaciones Clínicas ‘Norberto Quirno’ (CEMIC) qualified by the Department of Health and Human Services (HHS, USA): IRb00001745 - IORG 0001315.

### Mathematical model for voice production

#### Sound sources

The simplest way to achieve self-oscillations in the vocal folds during *voiced* sounds is changing the glottal shape over a cycle, giving rise to different pressure profiles that provide for the asymmetry needed to transfer mechanical energy to the folds and maintain their oscillation [20]. A simple dynamical system capturing the essentials of the flapping model has been developed and thoroughly studied in [10]. The equation of motion for the midpoint of the focal folds 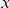 reads:
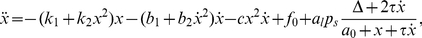
(1)where 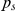 is the static sub-glottal pressure, 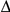 and 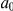 geometrical parameters of the glottal profile and 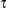 is the period of the convergent-divergent profile cycle of the vocal folds. The membrane tissue is described by a nonlinear restitution force of parameters 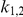 and a nonlinear dissipation of parameters 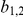 and 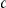. The pressure perturbation generated by this oscillation entering the vocal tract is 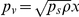, where 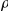 is the air density [8].

On the other hand, *unvoiced* sounds like whispering and fricative consonants are produced by turbulent sounds. Although there is no agreement about the acoustic mechanism generating frication, it is well established that turbulent sound is created as airflow is forced to go through a constriction, producing a colored noisy sound [4], [21]. As a raw approximation to this kind of sound source, we model the acoustic pressure 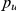 as a damped oscillator forced with white noise 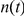,
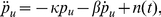
(2)such that the consonants sound spectra present a broad peak centered at 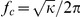 in the range 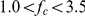 kHz and overall shape as reported in [11].

#### Vocal tract

The sound generated at the input of the vocal tract for voiced sounds or at a constriction in unvoiced sounds travels back and forth along a non-uniform vocal tract. We treat this tube as a concatenation of 10 short uniform tubes in which only plane wave-sound propagation is considered. This simplification is accurate for frequencies 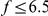 kHz [4], [11], which is consistent with the phonemes and noises analyzed here, whose spectral loads fall essentially within that frequency range (see figure 4). The 10 tube approximation represents a compromise between computational effort and good resolution for the vocal tract shape.

The boundary conditions for the pressure at the tube interfaces read:
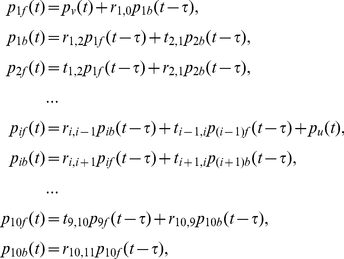
(3)where 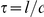 is the propagation time of the sound in a tube of length 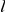, and 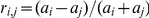 and 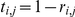 are the reflection and transmission coefficients for the sound wave at the interface between successive tubes. In particular, 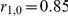 is the reflection coefficient at the entrance of the vocal tract (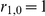 for a closed tube), and 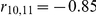 is the reflection coefficient at the vocal tract exit (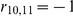 for an open tube). Equations 3 consider both the voiced sound source produced by the vocal folds (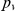, eq. 1) and unvoiced case, (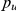, eq. 2) after a constriction in the 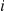
th tube.

The complete model of equations 1 and 3 for voiced sounds and 2 and 3 for unvoiced sounds allows synthesizing speech sounds from a set of anatomical parameters, 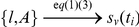 and 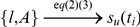. However, in this work we deal with the opposite task, i.e. finding the best vocal anatomy approximating an experimental sound spectrum. The main obstacle to accomplish this task is the dimension of the parameter space, proportional to the number of tubes approximating the vocal tract. In our case, the 11-dimensional parameter space 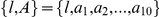 is investigated using a genetic algorithm.

#### Genetic algorithm

A genetic algorithm is an optimization procedure inspired by natural selection. The rough idea behind natural selection is that the best adapted individuals of a species contain good genetic blocks. These individuals prevail in reproduction, generating offspring that exploit those blocks by two processes: by mixing the genetic information of their parents (crossover) and by local random changes (mutation). The application of these two operators is a very efficient way to explore the genetic space of the population in search for new, better adapted individuals [22].

This caricature can be exported to find the set of anatomical parameters that best reproduce a given experimental sound spectrum 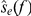 (target spectrum) as follows:

- we associate a fitness function 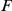 to the parameter set 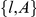 by computing the synthetic sound 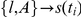, finding its Fourier transform 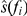 and calculating the inverse of the square error between the experimental and the synthetic spectra, 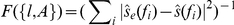, 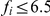 kHz. In the case of including the anatomical constraints, we used 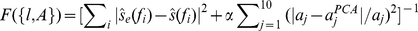 for a vocal tract of areas 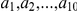. The factor 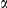 is set to generate a relative weight of 40% for the anatomical constraints and 60% for the spectral properties.
- We associate a genetic space to each parameter 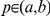 by normalizing it 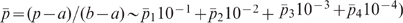 and associating it to the string 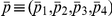.
- The n-dimensional set 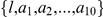 is replaced by the 4n-dimensional string 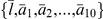. In this space, the crossover operator is just an interchanging of the elements of two of these strings at a random location. In turn, the mutation operator is just the replacement of a given element of the string by another in a random location.

The algorithm starts with a random population 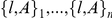 of 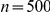 vocal tracts, from which 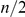 pairs are selected with a probability proportional to their fitness 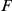. For each pair, crossing over and mutation occur with probabilities of 80% and 10% respectively. The resulting pairs constitute the new population of vocal tracts, and the process continues until 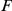 reaches some desired threshold.

In this way, after 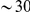 recursions, the algorithm typically produces at least 10% of vocal tracts whose spectral square differences with respect to the target spectrum are below the 5% of the total spectral power.

Throughout this work, we specifically:

- use an average over 10 sound spectra (for vowels, fricatives, clicks and knocks in each experiment) as the target spectrum;
- we penalize abrupt shape variations by making the fitness function proportional to 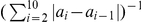, therefore obtaining smooth results.
- In all the figures, we show the average of the vocal tracts whose spectra are within the 5% difference with respect to the experimental.

### Natural sounds

In order to characterize the spectra of the knock and click sounds, we built a database of recording samples of knocking on different doors and desks in similar conditions, i.e. avoiding the presence of echoes, at 1 m distance and sampling rate of 44 kHz. For the clicks, we recorded samples of the noises produced by pressing on different computer mouse buttons and light switches. In each case, we selected 20 samples, calculated the spectra and normalized them. Every spectrum presented a similar frequency range, and similar relevant features concentrated in 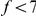 kHz. The averaged click and knock spectra are presented in figure 4.

### Experiments

#### Experimental procedure for vowels

In this experiment, 20 subjects were asked to associate a vowel to each of 5 audio files, played in random order, in a non-forced-choice paradigm. Audio files were generated synthesizing 1 s of sound using the following source parameters for equation 1: 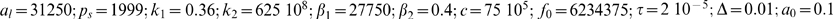. The resulting time series were injected into the vocal tracts of figure 2 (table 2) and then normalized and converted to wav files (available at Supporting Information, Audio S1, S2, S3, S4 and S5 for the Spanish [a,e,i,o,u] respectively). In this way, every sound was synthesized with the same pitch 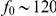 Hz and timbre, and therefore the acoustic differences correspond exclusively to the vocal tract anatomy.

All the participants listened to audio files at 1 m distance of the loudspeakes, connected to a PC in a silent room and filled a sheet of paper indicating the chosen vowel for each audio file. Results are summarized in table 1.

#### Experimental procedure for fricatives and onomatopoeia. First experiment

For this experiment we used recordings of 5 real coarticulated consonants 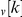. The original files consisted of recordings of the syllables 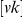 for the set 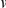 of 5 Spanish vowels. These audio files were edited and the vowel parts cutted out. This procedure is straightforward, because in normal speech the vowel and consonant are naturally isolated from each other, as shown in the knock or click time series, upper panels of figure 4. Finally, the sound intensity was normalized. With this procedure we generated a pool of 4 sets of the 5 coarticulated consonants from from 2 male and 2 female speakers. (two sets of experimental samples are available at Supporting Information, Audio S6, S7, S8, S9, S10 and S11 a S15 ordered as 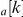, 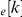, 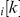, 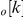 and 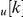 for each set).

A total of 20 participants performed the experiment, divided in 2 different sessions. The order of the sessions was randomized. In both of them they listened to a set of coarticulated consonants, chosen at random. In one session, we asked the participants to grade the similitude of each file with respect to their own representation of a strike on a door. In another session, the instruction was to grade the similitude of the sound files with respect to their idea of the sound produced by pressing on a mouse button.

All the participants listened to audio files at 1 m distance of the loudspeakes, connected to a PC in a silent room and filled a sheet of paper indicating the grade for each sound file, using a scale from 1 (no association with the instructed sound) to 10 (perfect identification with the instructed sound).

#### Second experiment

For this experiment we used 7 sound files. We synthesized sound for the the 5 reconstructed fricatives 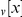 of figure 3 and for the optimal vocal tracts for the click and knock sounds without anatomical restrictions (figure 4). The parameters of the sound source are detailed in the captions of figure 3 and 4, and the vocal tract parameters in table 2. Every time series was multiplied by the envelope of an experimental 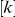 of 30 ms duration, and converted into a wav file (see Supporting Information, Audio S16, S17, S18, S19 and S20 for the synthetic 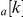, 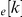, 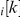, 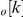 and 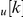 respectively, Audio S21 and S22 for the optimal knock and click).

This experiment was performed by another set of 20 participants, using the same procedure as for the first experiment. Participants listened to the set of consonants selected at random and graded them in a sheet of paper.

Every participant declared to have a well formed idea of both types of sounds (blowing on a door and pressing a computer mouse button) to use them as a reference in grading the sound files presented. The results of both experiments are summarized in figure 5, were the average grades and standard deviations are shown. Dotted lines correspond to grading the consonants with respect to the sound of a light switch/computer mouse button, and solid lines to the strike on a door.

## Supporting Information

Audio S1Synthetic Spanish vowel 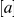 (wav format).(WAV)

Audio S2Synthetic Spanish vowel 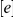 (wav format).(WAV)

Audio S3Synthetic Spanish vowel 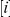 (wav format).(WAV)

Audio S4Synthetic Spanish vowel 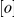 (wav format).(WAV)

Audio S5Synthetic Spanish vowel 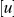 (wav format).(WAV)

Audio S6Experimental coarticulated consonant (wav format) 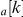, set 1.(WAV)

Audio S7Experimental coarticulated consonant (wav format) 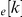, set 1.(WAV)

Audio S8Experimental coarticulated consonant (wav format) 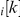, set 1.(WAV)

Audio S9Experimental coarticulated consonant (wav format) 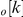, set 1.(WAV)

Audio S10Experimental coarticulated consonant (wav format) 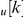, set 1.(WAV)

Audio S11Experimental coarticulated consonant (wav format) 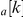, set 2.(WAV)

Audio S12Experimental coarticulated consonant (wav format) 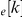, set 2.(WAV)

Audio S13Experimental coarticulated consonant (wav format) 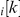, set 2.(WAV)

Audio S14Experimental coarticulated consonant (wav format) 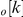, set 2.(WAV)

Audio S15Experimental coarticulated consonant (wav format) 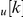, set 2.(WAV)

Audio S16Synthetic coarticulated consonant (wav format) 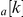.(WAV)

Audio S17Synthetic coarticulated consonant (wav format) 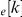.(WAV)

Audio S18Synthetic coarticulated consonant (wav format) 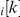.(WAV)

Audio S19Synthetic coarticulated consonant (wav format) 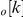.(WAV)

Audio S20Synthetic coarticulated consonant (wav format) 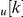.(WAV)

Audio S21 Synthetic sound of the optimal vocal configuration imitating the knock sound (wav format).
(WAV)

Audio S22 Synthetic sound of the optimal vocal configuration imitating the click sound (wav format).
(WAV)
